# Transfusion in Diabetic Coma

**Published:** 1899-09-02

**Authors:** 


### TRANSFUSION IN DIABETIC COMA.

There are few conditions of greater gravity than that of diabetic coma. So constantly is it fatal that it is generally regarded as a termination rather than as a complication of diabetes. Hence the importance of a line of treatment which gives any hope of even a temporary respite when this condition makes its appearance. About a year ago Dr. Thomas Oliver reported the case of a diabetic patient, who was admitted into the Newcastle-upon-Tyne Infirmary in a state of coma, for which he was successfully transfused. The patient was 30 years of age, and had then been suffering from diabetes for about eight months. He had rather rapidly developed polydipsia and polyuria, and in a few months had lost four stones in weight. His urine contained a large quantity of sugar. On July 12th, after having been treated by codeia and dieting, he left the infirmary to go home, feeling much better. That evening he was found in a railway carriage in a state of deep unconsciousness, and was brought back to the infirmary. On his admission he was transfused with two and a-half pints of a saline solution, and a purge was given. By degrees consciousness was regained, and since then, although he had not returned to his work, his life had been a fairly enjoyable one and he had gained weight, but had developed various nervous symptoms, probably indicating diabetic neuritis. Dr. Oliver now records[1] the sequel to this case. On March 12th of this year another attack of coma came on, and he died two hours after its onset. Dr. Oliver points out that there are physicians who do not believe in transfusing with saline fluid in diabetic coma, but, as he says, here was a case of a patient who lived 243 days after the operation, and ultimately died rapidly, apparently from a repetition of diabetic coma, having in the meantime enjoyed a condition of health which, had he been a wealthy man, would have enabled him to arrange his affairs, or to have disposed of his business.

[1] Lancet, Aug. 26.

